# A Boundary-Information-Based Oversampling Approach to Improve Learning Performance for Imbalanced Datasets

**DOI:** 10.3390/e24030322

**Published:** 2022-02-23

**Authors:** Der-Chiang Li, Qi-Shi Shi, Yao-San Lin, Liang-Sian Lin

**Affiliations:** 1 Department of Industrial and Information Management, National Cheng Kung University, University Road, Tainan 70101, Taiwan; lidc@mail.ncku.edu.tw (D.-C.L.); r36091050@gs.ncku.edu.tw (Q.-S.S.); 2 Singapore Centre for Chinese Language, Nanyang Technological University, Ghim Moh Road, Singapore 279623, Singapore; yao-san.lin@sccl.sg; 3 Department of Information Management, National Taipei University of Nursing and Health Sciences, Ming-te Road, Taipei 112303, Taiwan

**Keywords:** boundary information, synthetic sample generation, imbalanced datasets

## Abstract

Oversampling is the most popular data preprocessing technique. It makes traditional classifiers available for learning from imbalanced data. Through an overall review of oversampling techniques (oversamplers), we find that some of them can be regarded as danger-information-based oversamplers (DIBOs) that create samples near danger areas to make it possible for these positive examples to be correctly classified, and others are safe-information-based oversamplers (SIBOs) that create samples near safe areas to increase the correct rate of predicted positive values. However, DIBOs cause misclassification of too many negative examples in the overlapped areas, and SIBOs cause incorrect classification of too many borderline positive examples. Based on their advantages and disadvantages, a boundary-information-based oversampler (BIBO) is proposed. First, a concept of boundary information that considers safe information and dangerous information at the same time is proposed that makes created samples near decision boundaries. The experimental results show that DIBOs and BIBO perform better than SIBOs on the basic metrics of recall and negative class precision; SIBOs and BIBO perform better than DIBOs on the basic metrics for specificity and positive class precision, and BIBO is better than both of DIBOs and SIBOs in terms of integrated metrics.

## 1. Introduction

Data is said to be imbalanced when one of its classes (majority class, negative class) has many more examples than that of other classes (minority class, positive class). This occurs in many real-world cases, such as customer credit risk prediction [1], bankruptcy prediction [2], product fault diagnosis [3], medical data analyses [4], fraud prediction [5], etc. In these cases, their minority class is typically interesting, important, and has high misclassification costs. However, when traditional classifiers are used to classify them, the classifications usually bias the majority class. This paper calls these unsatisfactory learning results for imbalanced data learning problems.

Many studies have shown the reasons for imbalanced data learning problems including: (1) a high imbalance ratio, where the misclassifications of positive examples are regarded as tolerable errors because the total classification accuracy is high enough even though the examples are classified as negative, (2) a small disjuncts problem [6,7], where a small number of positive examples forming subclusters that cannot be ignored are usually misclassified to reduce model complexity, (3) overlapping [8,9], which is an area that includes both the majority class and the minority class. Positive examples in the overlapping are typically sacrificed in order to minimize structural risk.

To improve the performance of learning from imbalanced data, many kinds of methods have been proposed. This paper divides them into five classes as follows:

(1) *Algorithmic modification*. Some traditional classifiers work well on imbalanced data after their internal operations are changed. Yu et al. [10] utilized an optimized decision threshold adjustment strategy in a support vector machine (SVM). Zhao et al. [11] proposed a weighted maximum margin criterion to optimize the data-dependent kernel in an SVM. In addition to kernel-based SVMs, fuzzy rule-based classification systems are used and modified to deal with imbalanced data, such as in López et al. [12] and Alshomrani et al. [13].
(2) *Cost-sensitive classification*. This classification takes into consideration that the minority class misclassification costs are more expensive than those for the majority class. Zhou and Liu [14] moved an output threshold toward majority class such that minority class examples become more difficult to misclassify in a training cost-sensitive neural network. Siers and Islam [15] proposed a cost-sensitive voting technique to minimize the classification costs for a decision forest. Lee et al. [16] adjusted factor scores by categorizing instances based on an SVM’s margin for AdaBoost.
(3) *Ensemble learning*. This class can be used reduce variances by aggregating predictions of a set of base classifiers. Sun et al. [17] investigated cost-sensitive boosting algorithms with different weight updating strategies for imbalanced data. Sun et al. [18] turned an imbalanced dataset into multiple balanced sub-datasets and used them in base classifiers. Another very common way type of ensemble learning is where it is combined with resampling techniques, such as SMOTEBagging [19], random balance-boost [20], and the synthetic oversampling ensemble [21].
(4) *Data particle geometrical divide (GD)*. The GD technique creates class-based data particles to classify data examples by comparing data gravitation between different data particles. Rybak and Dudczyk [22] developed a new GD method with four algorithms for determining the mass of a data particle to effectively improve gravitational classification in the Moons and Circles datasets. Furthermore, Rybak and Dudczyk [23] proposed the variant of GD method named unequal geometrical divide to improve classification performance of imbalanced occupancy detection datasets.
(5) *Resampling techniques*. Here, the aim is to balance the class distribution by removing majority class examples (undersampling) or by inflating minority class examples (oversampling). Since the synthetic minority oversampling technique (SMOTE) [24] was proposed in 2002, it has become one of the most influential data preprocessing/oversampling techniques in machine learning and data mining. To improve the SMOTE, undersampling techniques e.g., condensed nearest neighbor (CNN) [25], Tome lines [26], etc. were used after the oversampling. SMOTE_IPE [27] is another combined resampling method. It uses an iterative-partitioning filter [28] to remove noisy samples in both majority and minority classes to clean up boundaries and make them more regular. Li et al. [29] used the mega-trend-diffusion technique [30] for undersampling and used a two-parameter Weibull distribution estimation for oversampling in their work. A more improved oversampling technique will be introduced in Section 2.

Among the above referenced techniques, resampling is the most common for handling imbalanced data since it can be regarded as preprocessing of the previous three techniques, and it is simple-to-use do to not involving complex classifier algorithms. Instead of undersampling, which may result in discarding useful data that worsen variances and produces warped posterior probabilities [31], developing oversampling techniques (or oversamplers) has attracted more attention. The next section introduces more oversamplers. In Section 3, their advantages and shortcomings are discussed, and the motivation of the study is provided. Then, our method using boundary information is proposed in Section 4. In Section 5, two experiments are designed to provide strength comparisons of different oversamplers and the performance verifications of the proposed method. The experimental results are shown and discussed in Section 6, and conclusions are drawn in Section 7.

## 2. Oversampling Techniques

More oversampling techniques are introduced in this section. For convenience, suppose ***m*** is a majority class example selected to be oversampled; ***p*** (or ***n***) is a positive (or negative) example selected to be oversampled with ***m***, and ***s*** is a synthetic sample generated by ***m*** and ***p*** (or ***n***).

The simplest method for oversampling is random oversampling (ROS). Because it is sampling with replacement, each sample can be trained at least two times using classifiers, which causes overfitting. Chawla et al. [24] suggested that ROS makes classifiers learn specific patterns. Instead of duplication, SMOTE created ***s*** by s=m+r×(p−m), where ***p*** was a random one of the *k* nearest positive neighbors (*k*NPN) of ***m***, and *r* was a real value between 0 and 1. Because s was different from ***m*** and ***p***, it was called a synthetic sample. Synthetic samples can help classifiers create more general patterns.

To generate more helpful synthetic samples, some studies have suggested that dangerous minority class examples are more important and that new samples should be created dependent on them. Han et al. [32] regarded ***m*** as dangerous if at least half of its *k* nearest neighbors (*k*NNs, containing both minority class and majority classes are majority class examples. Then, only dangers were selected to be oversampled by using SMOTE. This procedure is called Borderline-SMOTE1 (B1_SMOTE). If ***s*** was not only created between two dangerous examples, but also between a dangerous example (***m***) and its *k* nearest negative neighbor (*k*NNN, ***n***), the approach is called Borderline-SMOTE2 (B2_SMOTE), where ***s*** is computed using the formula s=m+r×(n−m), and ***r*** is a real value between 0 and 0.5, so that ***s*** is closer to ***m***. Similarly, the adaptive synthetic sampling approach (ANASYN) [33] deems that harder-to-learn examples are more important. It defines the difficulty level of learning ***m*** by the ratio of the number of majority classes to the number of minority classes in the *k*NNs of ***m***. Then, examples with greater difficulty levels are more easily oversampled using SMOTE. Instead of using the counts to determine important examples, borderline over-sampling (BOS) [34] and synthetic informative minority over-sampling (SIMO) [35] identify dangerous examples based on decision boundaries trained by an SVM. Then, BOS generates synthetic samples by using interpolation or extrapolation techniques based on the ratio of the majority class to the minority class; SIMO generates synthetic samples based on the distance to decision boundaries, and the examples being misclassified by the SVM are thus more likely to be oversampled. The majority weighted minority oversampling technique (MWMOTE) [36] was designed as a new approach to determine boundary examples. Initially, the majority set near the minority set was considered to be the borderline majority class. Second, the minority set near the borderline majority set was considered to be the borderline minority class. Then, the denser the majority set was, and the sparser the minority set was, the more important the borderline examples were. In the latest approaches, an attribute weighted *k*NN hub on SMOTE (AWH_SMOTE) [37] was applied to the *k*NN hub to find informative examples. Examples with rare occurrences in *k*NN hub were considered to be more dangerous and thus more important.

Conversely, other oversamplers considers safe minority class examples to be more important, and thus, it is felt that new samples should be created based on them. Safe-level-SMOTE (SL_SMOTE) [38] attempts to generate synthetic samples in safe regions. It defines the safe level of ***m*** by using the number of minority class in its *k*NNs. Let *slm* be the safe level of ***m***, and *slp* be the safe level of ***p***. If *slm* is larger than *slp*, then the s generated between ***m*** and ***p*** is positioned near ***m***, and vice versa. However, when ***m*** and ***p*** are from two different subgroups, ***s*** will fall into a majority class group to become noise. The local neighborhood extension of SMOTE (LN_SMOTE) [39] fixed this problem by selecting oversampled examples from their *k*NNs rather than from their *k*NPN. Instead of using *k*NNs, cluster-SMOTE (C_SMOTE), proposed by Cieslak et al. [40], clusters the minority class first, then selects oversampled examples in the same clusters. In addition, synthetic oversampling of instances by clustering and jittering (SOI_CJ) [41] utilize jittering process within the same clusters so that only one example is selected to be oversampled each time. Douzas et al. [42] proposed the k-means SMOTE (km_SMOTE), which uses a k-means algorithm to cluster the entire dataset; then, only the clusters dominated by the minority class can be used to oversample, where the sparser the clusters are, the more synthetic samples the clusters generate.

**Figure 1:**
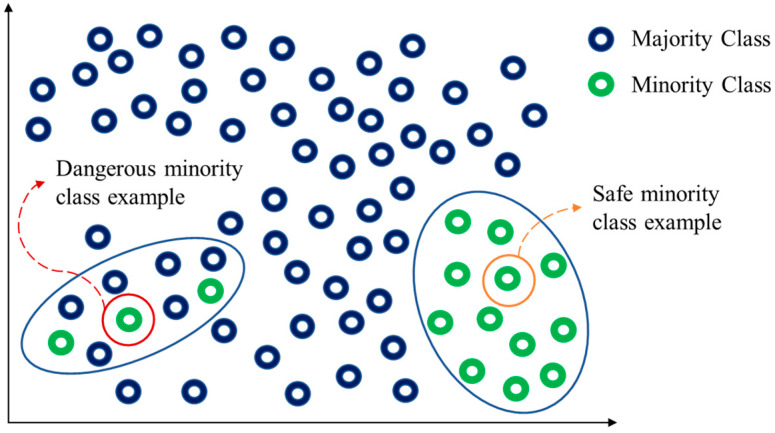
Two types of majority class examples.

To be brief, when a minority class example is surrounded by most of majority class examples, it is called the dangerous minority class example. On the contrary, it is called the safe minority class example as shown in Figure 1.

## 3. Motivation

From the overall reviews of the oversampling techniques in Section 2, we find that some oversamplers suggest that the important minority class examples are those that are dangerous, borderline, hard to learn, and misclassified, whereas others suggest that safe examples are the most important. When oversamplers based on dangerous minority class examples to create samples, oversamplers were called danger-information-based oversamplers (DIBOs). Conversely, based on safe ones, others were called safe-information-based oversamplers (SIBOs). We summarized oversamplers in Table 1, respectively.

**Figure 2:**
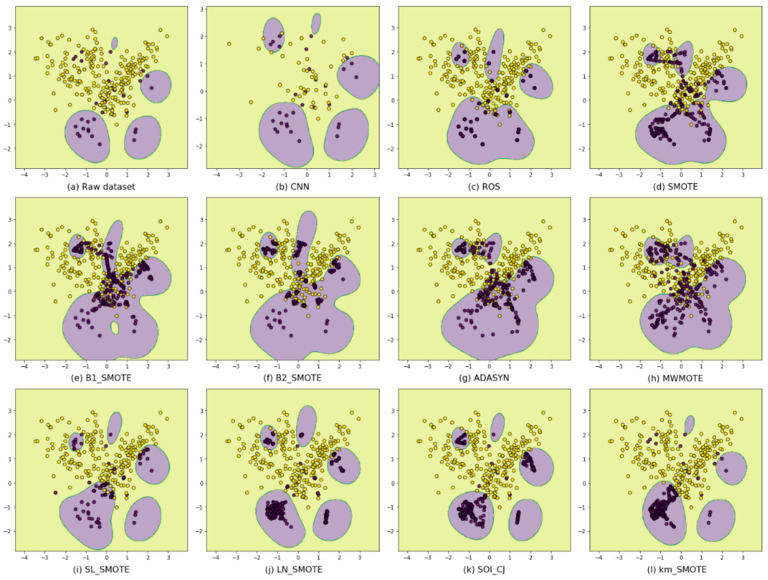
Classification results using different resamplers. (**a**) the raw imblanced dataset; (**b**) using CNN; (**c**) using ROS; (**d**) uisng SMOTE; (**e**–**h**) using danger-information-based oversamplers; (**i**–**l**) using safe-information-based oversamplers.

We find that DIBOs generate synthetic samples biased toward majority class areas, which can strengthen the decision boundaries necessary to be correctly classified as minority class. SIBOs generate synthetic samples biases toward minority class areas, which can protect safe regions from being misclassified as majority class. However, the extra samples generated by DIBOs become noises that affect the classification of the majority class, while SIBOs tend to ignore the minority class borderline examples. To visually understand these, a makeup imbalanced dataset shown in Figure 2a is taken to be resampled to balance classes using different methods, and then they are classified by using SVM classifiers with the same parameter settings. As can be seen in Figure 2e–h, the synthetic samples generated by DIBOs are more radical, so the predicted decision boundaries are biased toward the majority class. As shown in Figure 2i–l, the synthetic samples generated by SIBOs are more conservative, so fewer majority class examples are misclassified, while at the same time, more borderline minority class examples are not being correctly classified.

Based on the advantages and disadvantages of DIBOs and SIBOs respectively, this paper proposes a new boundary information concept, where the created samples depending on it are close to the decision boundaries, rather than close to the more dangerous areas as with DIBOs or close to the safer areas as with SIBOs.

## 4. Methodology

In this section, this paper firstly defines the boundary information; then, this section introduces the procedure for a boundary-information-based oversampler (BIBO), after which we provide an analysis of its strengths.

### 4.1. Boundary Information

In MWMOTE [36], the Euclidean distance is used to compute the information weight for minority class examples. Euclidean distance is also used in computing the similarity of two points. As in a similarity calculation, this paper claims that the information weight (*IW*) of ***b*** on ***a*** denoted by IW(a←b) is negative to the Euclidean distance from ***b*** to ***a*** denoted by ‖b−a‖ and that is exponential decay by ‖b−a‖ as Equation (1):

(1) IW(a←b)=e(−‖b−a‖),

Suppose in a minority class example ***m*** that its *IW* is very small if another example is far away from it. Therefore, we can only consider the IWs between ***m*** and its *k*NNs. Suppose that $minkNNs=\left\{p_{1},p_{2},\ldots ,p_{i}\right\}$ are *k*NPNs in the *k*NNs of ***m***, then $IW\left(m\leftarrow minkNNs\right)=\sum_{1}^{i}e\left(-\left\|p_{i’}-m\right\|\right)$; $majkNNs=\left\{n_{1},n_{2},\ldots ,n_{j}\right\}$ are *k*NNNs in the *k*NNs of ***m***, and $IW\left(m\leftarrow majkNNs\right)=\sum_{1}^{j}e\left(-\left\|n_{j’}-m\right\|\right)$, where i+j=k. Obviously, compared with IW(m←majkNNs), the larger IW(m←minkNNs) is, the safer ***m*** is, and vice versa. Thus, this paper calls the safe information weight (SIW) of ***m*** and call IW(m←majkNNs) the danger information weight (DIW). It can be said that the virtual samples generated by DIBOs are biased towards dangerous examples and that the SIBOs generate new samples biased towards safe examples. As discussed in Section 3, this paper suggests that created samples should be biased towards decision boundaries.

**Figure 3:**
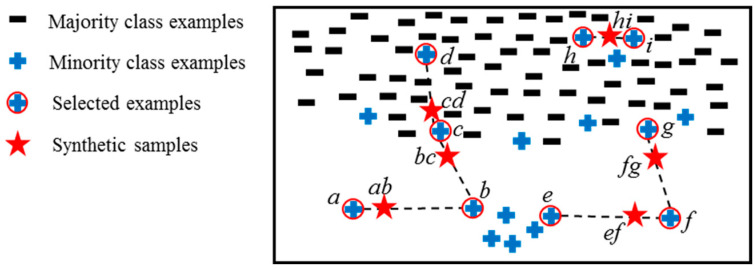
A demonstration of created samples that are biased towards the larger *BIW*.

As a rule of thumb, examples having as much DIW and SIW are more likely to be decision points. To find desirable decision boundaries, this paper defines a new concept of boundary information (BI), and the BI weight (*BIW*) is computed using Equation (2):

(2) BIW(m)=IW(m←minkNNs)×IW(m←majkNNs),

From Equation (2), it is known that an example has zero *BIW* when all of its *k*NNs are minority class, which this paper calls a redundancy; an example also has zero *BIW* when all of its *k*NNs are majority class, which this paper calls noise; an example has great *BIW* only when its DIW and SIW are both large. This paper proposes that synthetic samples should be biased towards examples with larger *BIW*, and Figure 3 is used to demonstrate the expected effects of this assumption. For example, the *ab* and *bc* are far away from *b* since the *b* is a safe example with very low *BIW*; the *bc* and *cd* are near *c* since *c* is a decision point with great *BIW*; the *cd* is far away from *d* since *d* is a noise; the *ef* is closer to *f* as compared to *e* since the *BIW* of *f* is larger than the *BIW* of *e*; in the case of small disjunct examples *h* and *i*, their created sample *hi* is within them so it is easier for them to be recognized.

### 4.2. Procedure for the Boundary-Information-Based Oversampler

This paper calls the oversampler that generates synthetic samples near the examples having larger *BIW*, the boundary-information-based oversampler (BIBO). The procedure for the BIBO is proposed in Table 2.

### 4.3. Computational Complexity of BIBO

The computational complexity of the proposed BIBO algorithm depends on the number of major class examples *N*, the number of original imbData *n*, and the number of minority class examples *P*. In Table 2, the for loop (*p* in *P*) indicates that we perform *P* times of calculations of *BIW* and r with each synthetic sample generation. In our algorithm, the number of synthetic samples is set as 2*N* − *n*. Namely, when the size of imbData is increased from n to 2*N*, the proposed BIBO algorithm is stopped. Therefore, the computational complexity of the BIBO algorithm can be calculated by Equation (3):

(3) $$Computational\;Complexity_{\mathrm{BIBO}}=O\left(P(2N-n)\right)$$

### 4.4. Strengths Analysis

The proposed BIBO selects minority class examples to be oversampled for which the BIWs are not zero in order to filter out examples with noise and redundancy. Then, the created samples are far away from both safe examples and dangerous examples, and they are closer to boundary examples. Also, it is easily understood. Only two parameters are considered. The capital letter *K* is used for the purpose of determining the *k*NPNs for oversampling, and the small letter *k* is used for determining the *k*NNs for the purpose of computing BIWs.

**Figure 4:**
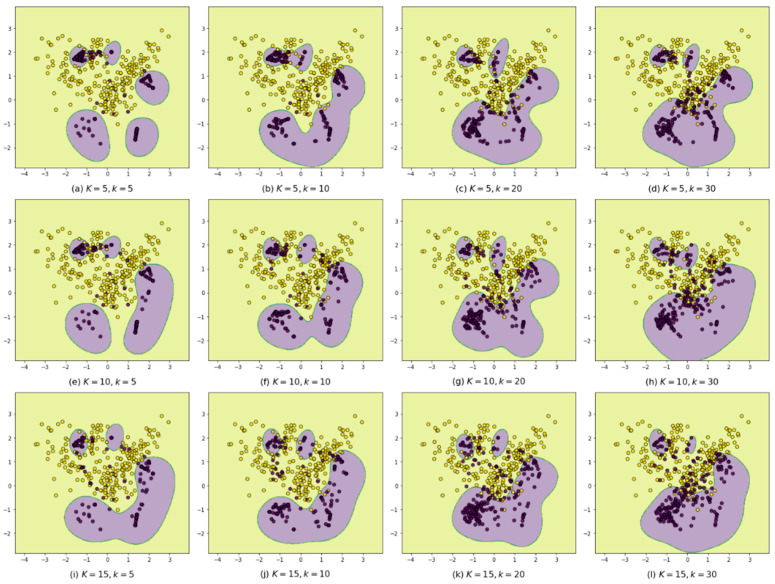
The classification results using BIBOs with different values of *K* and *k*.

To illustrate the strengths of the proposed BIBO, the imbalanced dataset shown in Figure 2a is applied to be oversampled by using the BIBOs with different values of *K* and *k*. Then, they are trained and classified using the same SVM classifiers. The classification results are shown in Figure 4, where from top to bottom, the *K* values increase from 5 to 15, and from left to right, the *k* values increase from 5 to 30. From the figures, this paper finds that when both the *K* and *k* are small (see Figure 4a), the BIBO is conducted like SIBOs; when the *K* increases from 5 to 15, the areas predicted to be positive (PPAs) are larger, and some separated PPAs are merging, as the *k*NPNs become larger and generate virtual samples inside them; when the *k* increases from 5 to 30, the PPAs become larger and start to intrude into the majority class areas as the old noises in the overlapping areas become fewer in number. However, even though both *K* and *k* are large enough, as shown in Figure 4l, the synthetic samples are still near the decision boundaries. Therefore, the BIBO has a great tolerance for parameter value settings.

## 5. Experiment

The experiment designs weighing the comparative strengths of the various oversamplers and the performance verification of the BIBO are introduced in this section. Before introducing them, this section provides the results of the classification evaluation metrics and the oversampler evaluation procedure, respectively.

### 5.1. Evaluation Metrics

Accuracy rate (*acc*) is the common metric for evaluating classifications, for which Equation (4) is the formula. However, using *acc* is cause of imbalanced data learning problems because it creates biases toward the majority class, as mentioned in Section 1. To balance the effects of two classes, the confusion matrices shown in Table 3 are used to formulate the imbalanced data classification evaluation metrics. The recall (*rec*) calculated using Equation (5) and the specificity (*spec*) calculated using Equation (6) are the true positive rate and true negative rate, respectively, that is to say, the percentages of correct classifications of the classes. The positive class ($pre_{P}$) precision calculated using Equation (7) and the negative class ($pre_{N}$) precision calculated using Equation (8) are the positive predictive value and the negative predictive value, respectively, in other words, the correct rates for the predicted values. This section calls these the five basic metrics, and the metrics integrated by two or more basic metrics are called integrated metrics.

Considering *rec* and *spec*, *g*-measure (*Gmean*) is defined as the geometric mean of *rec* and *spec*, which is calculated using Equation (9). Instead of considering only proportions that are being correctly classified, the *F*-measure (*Fmeas*) takes $pre_{P}$ into account and is calculated using Equation (10). Another well-known measure is the area under the ROC curve (AUC) [43]. In the ROC chart, the x-axis is 1-*spec*, and the y-axis is *rec*, and the curve shows their tradeoff by giving a decision cut-off. Obviously, these five are integrated metrics, in which the β in *Fmeas* is set as 1 in our experiments:

(4) $$acc=\frac{TP+TN}{TP+FP+FN+TN}$$

(5) $$rec=\frac{TP}{TP+FN}$$

(6) $$spec=\frac{TN}{TN+FP}$$

(7) $$pre_{P}=\frac{TP}{TP+FP}$$

(8) $$pre_{N}=\frac{TN}{FN+TN}$$

(9) $$Gmean=\sqrt{rec\times spec}$$

(10) $$Fmeas={\left(1+\beta \right)}^{2}\times rec\times \frac{pre_{P}}{\beta^{2}\times rec+pre_{P}}$$

### 5.2. Dataset Description

To verify the universality of the oversamplers, this paper tests some datasets that are downloaded from the KEEL-dataset repository [44]. Because the differences in the MM-metrics on different datasets are not commensurate, this paper uses the rankings of the oversamplers on each dataset to obtain their mean. Then, the mean rankings can be regarded as the performance measures of the oversamplers on the classifier.

#### 5.2.1. The Simulated Datasets

A set of 2-dimensional datasets [45] that are simulated by using different values of number of examples (Ex.), the imbalance ratio (IR), and the disturbance ratio (DR) are used in this experiment; see Table 4, in which the paw that its minority class is decomposed into three elliptical subregions that resemble a paw print; the clover that is its minority class resembles a flower with five elliptical petals; in the subcl, there are five small disjuncts shaped like rectangles.

#### 5.2.2. The Real-World Datasets

Oversamplers using the *k*NN concept are not applicable when dealing with highly imbalanced datasets because most minority class examples in them would be recognized as noise and lead to the wrong results. Therefore, the real-world datasets with imbalance ratios between 1.5 and 9 used in Fernández et al. [46] are used in this experiment. In addition, the ionosphere dataset downloaded from the UCI machine learning repository [47] is used in our experiment. The dataset has 17 pulse numbers with two attributes and one output to indicate returns of electromagnetic signals. Moreover, one big dataset named Swarm Behaviour Aligned with 2400 attributes and 24,017 samples downloaded from UCI machine learning repository is used in our experiments. Thus, a total of 23 datasets are used to implement our experiments. They are shown in Table 5, where Att. is the number of attributes.

### 5.3. Oversampler Performance Evalutation

This paper uses the *k*-fold cross-validation procedure to obtain the performance measures of oversamplers on every metric. For an imbalanced dataset and its one cross-validation process, first, the data set is partitioned into a training set and a testing set. Second, the training set is oversampled using an oversampler. Third, a classifier is trained using the oversampled set. Fourth, the testing set is used on the trained classifier to obtain the evaluation metrics. This process is repeated *k* times to obtain the mean of the metrics. Since the oversampler can increase the variances in the classifier, this paper further repeats the *k*-fold-cross-validation process *K* times to obtain the mean of the mean metrics (MM-metrics). Then, the MM-metrics can be regarded as the performance measures of the oversampler on both the dataset and the classifier.

Based on the above process, this paper uses different oversamplers containing RAW (without oversampler), SMOTE, B1_SMOTE, B2_SMOTE, ADASYN, MWMOTE, SL_SMOTE, LN_SMOTE, SOI_CJ, km_SMOTE, and BIBO to obtain their MM-metrics, respectively. Among them, RAW is where the original training set is used without being oversampled; the B1_SMOTE, B2_SMOTE, ADASYN, and MWMOTE are DIBOs; the SL_SMOTE, LN_SMOTE, SOI_CJ, and km_SMOTE are SIBOs. These programs are imported from the smote_variants package [48], and their parameters are the default settings. The BIBO is programed as shown in Table 2, where the *K* and the *k* are set as 5 and 15, respectively. Then, these oversamplers are ranked based on their MM-metrics on every metric.

## 6. Results and Discussion

In this section, the two experiment results are introduced and discussed.

### 6.1. Comparative Strengths Results

As is known, some evaluation metrics contradict each other, such as *rec* and *spec*, $pre_{P}$ and $pre_{N}$. However, in most studies, only partial metrics were used to verify the effectiveness of their own proposed oversamplers. In this experiment, we attempt to apply all of the metrics to determine the comparative strengths of the different oversamplers. Since most oversamplers employ the concept of *k*NN, the *k*NN classifier is applied in this experiment. The comparative strengths of the oversamplers are presented using different metrics. The experimental results are shown in Table 6, and the findings are summarized as follows:

(1) All the oversamplers outperform the RAW on the *rec* and $pre_{N}$ basic metrics, but they do not on the *acc*, *spec*, and $pre_{P}$ metrics. This means that, for oversamplers, more positive examples are correctly classified, but at the same time, many more negative examples are incorrectly classified as positive values.
(2) The oversamplers outperform RAW on most of the integrated metrics and in terms of the average of all of the metrics (*ave*). This means that oversampling techniques are helpful for improving imbalanced data learning problems.
(3) Compared with SMOTE, DIBOs perform better on the *rec* and $pre_{N}$ basic metrics, and most SIBOs are better on the *acc*, *spec* and $pre_{P}$ metrics (with the exception of SL_SMOTE, which was caused by too many virtual samples being created in the negative class areas). It can be said that DIBOs tend to avoid missing any positive examples being correctly classified, and SIBOs improve the problem of the negative examples being incorrectly classified. However, DIBOs and SIBOs do not always outperform SMOTE on integrated metrics.
(4) On the *acc*, *spec*, and $pre_{P}$ basic metrics, BIBO has good performance like the SIBOs, and it outperforms all the DIBOs. On the contrary, for the *rec* and $pre_{N}$ metrics, BIBO outperforms all the SIBOs, similar to the DIBOs. These findings confirm that BIBO is better than the SIBOs and DIBOs in general due to moving virtual samples toward decision boundaries.
(5) BIBO has better performance results on the *Fmeas*, *AUC* and *ave*. Hence, BIBO is better oversampler for improving imbalanced dataset learning problems.

### 6.2. Performance Results

In this experiment, the most representative metrics containing *acc*, *Gmean*, *Fmeas*, *AUC*, and their average (*ave*) are used to measure the performance of oversamplers on the four classifiers *k*NN, C4.5, SVC_L, and SVC_S. This paper uses SVMs with linear and sigmoid kernel functions in which the C4.5 program [49] is a “DecisionTreeClassifier” with the “entropy” criterion imported from “sklearn.tree”; the SVM programs are “sklearn.svm. SVC” with “linear” and “sigmoid” kernels denoted as SVC_L and SVC_S, respectively. The results are shown in Table 7, and the findings are summarized as follows:

(1) When C4.5 is used as the classifier, BIBO obtains better results on all of the metrics, even the acc. It can be deduced that the virtual samples created by BIBO are near the real decision nodes on the decision tree.
(2) For the four classifiers and the five metrics on each of them, 20 metrics in total, half of them indicate that BIBOs have better performance results (the values in bold and underlined). Consequently, this further confirms that BIBO is better technique for improving imbalanced data learning problems.
(3) Some oversampler performance results are not better than those for RAW, especially in the case of SVC_L and SVC_S. This may have been caused by (1) contradictory metrics, (2) overlapping blurriness, (3) the noise of virtual samples, or (4) the effectiveness of some classifiers on some imbalanced datasets.

### 6.3. Comparative Results of Computational Complexity

In this paper, the test PC is equipped with an Intel^®^ Core^TM^ i7-10700 CPU @ 2.90 GHz and 32 GB RAM. The operation system is Ubuntu 20.04.2 LTS. A total of 23 datasets were used to perform comparisons of computational complexity between the proposed BIBO and nine algorithms. We sample 80 percent of data in each dataset to run 50 experiments. The experiments are implemented under above-mentioned environment with Python 3.8.10. The averages of computational time of the algorithms can be obtained as shown in Table 8. The SOI_CJ algorithm has the longest running time among them because it performs more computation on clustering in one big dataset, namely Swarm Behaviour Aligned, as shown in Table 5. The BIBO algorithm outperforms those of five algorithms on computational time.

### 6.4. An Example of Using the Proposed BIBO Method

In this section, we random draw 80 percent of data from ecoli-0_vs_1 dataset as an example to explain the proposed BIBO method in details. The data is set as a training dataset listed in. 

The implementation procedure of the BIBO method is explained in the following:

*Step 1*. The training dataset has 115 majority class examples (Positive) and 61 minority class examples (Negative) as shown in Table 9.

*Step 2*. Set K = 10 and k = 10 to compute values of *BIW* and r as shown in Table 2. We briefly listed the values of BIW(pp), BIW(p), and r in Table 10, respectively.

*Step 3*. Generate synthetic minority class examples as shown in Table 10.

*Step 4*. Stop the steps 1–3 when the number of training samples is twice of the number of majority class samples.

*Step 5*. Add generated synthetic examples into the original dataset to build up a balanced training dataset.

## 7. Conclusions

This paper defined the information weight (IW) between two points by using the reciprocal of a natural exponential function with the Euclidean distance as its index, where the total IWs of the minority (or majority) class examples in one’s *k*NNs is the safe (or danger) information weight (SIW, or DIW) in the example. Then, examples having larger SIWs (or DIWs) can be consider as safe (or danger). The comparison experiment proved that SIBOs generating synthetic samples near safe areas improves the performance of *spec* and $pre_{P}$ and that DIBOs generating synthetic samples near dangerous areas can improve the performance of *rec* and $pre_{N}$.

In the proposed oversampler (BIBO), the product of SIW and DIW is defined as the boundary information weight (BIW), where synthetic samples are generated near examples with larger BIWs. This indicates that the examples with both large SIWs and large DIWs are more likely to be decision points and that synthetic samples should be generated near them. The comparison experiment proved that BIBO has the advantages of both SIBOs and DIBOs. The performance verification experiment confirmed again that BIBO is better approach on the whole for handling imbalanced data learning problems. However, BIBO did not have the best performance in all cases. A more customized BIBO on different datasets or on different classifiers can be proposed in the future. In our future research, one can use other real datasets downloaded from UCI machine learning repository to verify the effectiveness of the customized BIBO. Another direction is to undertake verification using popular artificial neural network as learning models.

## Figures and Tables

**Table 1:** Two classes of oversamplers note apparent row misalignment.

Oversamplers	Authors [Reference]	Methods
Danger-information-based oversamplers (DIBOs)	Han et al. [32]	B1_SMOTE
		B2_SMOTE
	He et al. [33]	ADASYN
	Nguyen et al. [34]	BOS
	Barua et al. [36]	MWMOTE
	Piri et al. [35]	SIMO
	Fahrudin et al. [37]	AWH_SMOTE
Safe-information-based oversamplers (SIBOs)	Cieslak et al. [40]	C_SMOTE
	Bunkhumpornpat et al. [38]	SL_SMOTE
	Maciejewski and Stefanowski [39]	LN_SMOTE
	Sanchez et al. [41]	SOI_CJ
	Douzas et al. [42]	km_SMOTE

**Table 2:** The algorithm of the proposed boundary-information-based oversampler.

**Input:**	
imbData:	An imbalanced dataset.
*K*:	The number of kNPNs for oversampling.
*k*:	The number of kNNs for computing *BIW*.
**Output:**	
resData:	The imbData that had been resampled by this procedure
**Procedure Begin**	
1. P← minority class set from imbData	
2. N← majority class set from imbData	
3. resData = imbData	
4. while the length of resData < twice the length of *N*:	
5. for ***p*** in ***P***:	
6. KNNp← the *K* nearest neighbors of ***p*** from ***P***	
7. kNNp← the *k* nearest neighbors of ***p*** from imbData	
8. BIW(p)← computing *BIW* of ***p*** with ***kNNp***	
9. if BIW(p)=0: return to 4, then continue	
10. pp← randomly select an example from ***KNNp***	
11. kNNpp← the *k* nearest neighbors of ***pp*** from imbData	
12. BIW(pp)← computing *BIW* of ***pp*** with ***kNNpp***	
13. if BIW(pp)≥BIW(p):	
14. r=(BIW(pp)−BIW(p))/(BIW(pp)+BIW(p))	
15. s=p+(r,1)×(pp−p)	
16. else:	
17. r=2×BIW(pp)/(BIW(pp)+BIW(p))	
18. s=p+(0,r)×(pp−p)	
19. resData = resData + s:	
20. if the length of resData >= twice the length of ***N*** 21. break # the numbers of the two classes are balanced	
22. return the resData	
**Procedure End.**	

**Table 3:** Confusion matrix.

	Predicted Positive	Predicted Negative
Positive class	True positive (TP)	False Negative (FN)
Negative class	False positive (FP)	True Negative (TN)

**Table 4:** The simulated datasets.

No.	Name	Ex.	IR	DR (%)	No.	Name	Ex.	IR	DR (%)
1	paw1	600	5	0	10	clover4	800	7	0
2	paw2	600	5	30	11	clover5	800	7	30
3	paw3	600	5	60	12	clover6	800	7	60
4	paw4	800	7	0	13	subcl1	600	5	0
5	paw5	800	7	30	14	subcl2	600	5	30
6	paw6	800	7	60	15	subcl3	600	5	60
7	clover1	600	5	0	16	subcl4	800	7	0
8	clover2	600	5	30	17	subcl5	800	7	30
9	clover3	600	5	60	18	subcl6	800	7	60

**Table 5:** The real-world datasets.

No.	Name	Att.	Ex.	IR	No.	Name	Att.	Ex.	IR
1	ecoli-0_vs_1	7	220	1.86	12	page-blocks0	10	5472	8.79
2	ecoli1	7	336	3.36	13	pima	8	768	1.87
3	ecoli2	7	336	5.46	14	segment0	19	2308	6.02
4	ecoli3	7	336	8.6	15	vehicle0	18	846	3.25
5	glass-0-1-2-3 _vs_4-5-6	9	214	3.2	16	vehicle1	18	846	2.9
					17	vehicle2	18	846	2.88
6	glass0	9	214	2.06	18	vehicle3	18	846	2.99
7	glass1	9	214	1.82	19	wisconsin	9	683	1.86
8	glass6	9	214	6.38	20	yeast1	8	1484	2.46
9	haberman	3	306	2.78	21	yeast3	8	1484	8.1
10	new-thyroid1	5	215	5.14	22	ionosphere	34	351	1.79
11	new-thyroid2	5	215	5.14	23	Swarm Behaviour Aligned	2400	24017	2.20

**Table 6:** The rankings of the oversamplers using different metrics.

Oversamplers	Methods	*acc*	*rec*	*spec*	*Gmean*	$pre_{P}$	$pre_{N}$	*Fmeas*	*AUC*	*ave*
RAW	-	**4.09**	11.00	**2.52**	**5.57**	**2.39**	10.43	7.65	11.00	8.00
SMOTE	-	7.13	4.22	7.17	7.22	7.91	2.91	6.83	4.22	5.57
DIBOs	B1_SMOTE	7.30	5.30	7.87	7.26	**5.96**	4.00	6.26	5.30	6.80
	B2_SMOTE	10.00	7.78	9.96	10.22	9.65	6.35	9.91	7.78	9.91
	ADASYN	10.43	5.43	10.26	9.39	9.09	5.22	9.78	5.43	9.65
	MWMOTE	**6.70**	**1.57**	8.00	**5.91**	7.70	**2.04**	5.57	**1.57**	**5.26**
SIBOs	SL_SMOTE	9.17	8.96	7.22	9.74	9.04	8.22	9.87	8.96	9.87
	LN_SMOTE	**4.93**	**2.65**	**5.09**	**4.52**	**5.22**	**2.83**	**3.43**	**2.65**	**3.78**
	SOI_CJ	**3.02**	4.61	**4.04**	**2.83**	**4.00**	6.09	**2.04**	4.61	**2.04**
	km_SMOTE	**1.78**	4.91	**2.83**	**1.09**	**3.04**	8.09	**1.13**	4.91	**1.04**
BIBO	-	**1.43**	6.57	**2.04**	**2.26**	**1.00**	9.83	**3.52**	6.57	**3.30**

Note: The values in bold are the oversamplers that outperform the SMOTE (underlined ranking).

**Table 7:** The performance results of the oversamplers using different classifiers.

	**Classifiers**	***k*NN**					**C4.5**				
**Oversamplers**	**Methods**	***acc***	***Gmean***	***Fmeas***	***AUC***	***ave***	***acc***	***Gmean***	***Fmeas***	***AUC***	***ave***
RAW	-	4.65	6.52	8.35	8.00	8.96	7.61	8.09	8.57	9.52	8.26
SMOTE	-	7.59	8.04	7.61	5.83	7.30	7.39	7.13	7.09	7.00	7.04
DIBOs	B1_SMOTE	7.48	7.91	7.00	5.78	7.26	8.78	8.83	8.22	8.30	8.65
	B2_SMOTE	9.87	9.26	9.30	7.57	9.26	9.65	9.65	9.65	9.04	9.65
	ADASYN	9.17	7.43	7.57	3.74	7.04	6.30	5.35	4.61	2.87	4.30
	MWMOTE	6.20	5.39	5.26	**1.30**	5.00	4.48	3.61	2.48	4.78	3.25
SIBOs	SL_SMOTE	10.26	10.96	9.78	9.26	10.87	10.26	9.17	9.26	9.91	8.22
	LN_SMOTE	4.70	4.17	3.26	3.35	3.48	2.63	3.56	3.48	2.61	3.74
	SOI_CJ	2.65	2.35	**1.30**	3.04	**1.43**	2.57	3.04	4.13	4.39	4.13
	km_SMOTE	1.78	**1.30**	1.87	5.74	1.70	4.26	5.09	5.17	5.35	5.22
BIBO	-	**1.65**	2.65	3.70	6.39	3.70	**2.57**	**2.83**	**2.35**	**2.22**	**2.30**
	**Classifiers**	**SVC_L**					**SVC_S**				
**Oversamplers**	**Methods**	***acc***	***Gmean***	***Fmeas***	***AUC***	***ave***	***acc***	***Gmean***	***Fmeas***	***AUC***	***ave***
RAW	-	4.50	7.70	6.50	4.50	6.50	7.37	8.46	7.50	5.50	5.50
SMOTE	-	7.57	6.13	5.04	4.30	5.78	7.74	6.30	5.04	4.57	6.00
DIBOs	B1_SMOTE	8.96	9.46	6.87	6.48	7.13	8.91	7.70	6.70	6.22	7.09
	B2_SMOTE	9.96	8.70	7.87	7.39	7.96	9.00	8.61	7.83	7.17	8.00
	ADASYN	4.96	2.74	2.09	**1.61**	**2.26**	4.87	3.70	**1.83**	2.04	3.91
	MWMOTE	5.39	3.09	**1.96**	1.91	2.35	5.87	**2.39**	2.13	**1.35**	2.78
SIBOs	SL_SMOTE	7.54	10.46	10.50	10.50	10.50	7.37	10.46	10.50	10.50	10.50
	LN_SMOTE	5.00	5.35	4.91	5.26	5.13	4.48	5.30	5.00	5.70	5.00
	SOI_CJ	7.54	6.74	8.74	8.74	8.74	4.74	3.61	2.91	2.83	2.87
	km_SMOTE	2.76	**2.63**	4.70	6.61	2.57	2.70	2.53	4.87	6.39	**2.61**
BIBO	-	**1.83**	3.65	2.83	2.70	3.09	**1.96**	6.48	8.70	8.74	8.74

Note: The values in bold indicate the best ranking.

**Table 8:** The comparison of computational complexity.

**Oversamplers**	**SMOTE**	**B1_SMOTE**	**B2_SMOTE**	**ADASYN**	**MWMOTE**
computational time (s)	0.085	0.353	0.355	0.371	3.025
**Oversamplers**	**SL_SMOTE**	**LN_SMOTE**	**SOI_CJ**	**km_SMOTE**	**BIBO**
computational time (s)	0.899	2.002	50.090	4.076	0.689

**Table 9:** The training dataset.

NO.	Mcg	Gvh	Lip	Chg	Aac	Alm1	Alm2	Class
1	0.23	0.48	0.48	0.50	0.59	0.88	0.89	Negative
2	0.56	0.40	0.48	0.50	0.49	0.37	0.46	Positive
…	…	…	…	…	…	…	…	…
175	0.24	0.41	0.48	0.50	0.49	0.23	0.34	Positive
176	0.20	0.44	0.48	0.50	0.46	0.51	0.57	Positive

**Table 10:** Synthetic example generation.

No.	BIW(pp)	BIW(p)	r	Synthetic Examples
1	11.053	11.458	0.982	[0.50, 0.37, …, 0.69]
2	16.335	9.340	0.272	[0.00, 0.51, …, 0.44]
…	…	…	…	…
53	9.503	10.345	0.958	[0.12, 0.67, …, 0.63]
54	5.326	9.503	0.718	[0.33, 0.37, …, 0.65]

## Data Availability

Data available in a publicly accessible repository. The simulated datasets presented in this study are openly available at https://doi.org/10.1007/978-3-642-13529-3_18 (accessed on 22 February 2022), reference number [45]. The real-world datasets presented in this study are openly available at https://doi.org/10.1016/j.fss.2007.12.023 (accessed on 22 February 2022), reference number [46]. The UCI datasets presented in this study are openly available at reference number [47].
